# Antimicrobial Monomers for Polymeric Dental Restoratives: Cytotoxicity and Physicochemical Properties

**DOI:** 10.3390/jfb9010020

**Published:** 2018-02-27

**Authors:** Diane R. Bienek, Stanislav A. Frukhtbeyn, Anthony A. Giuseppetti, Ugochukwu C. Okeke, Drago Skrtic

**Affiliations:** Volpe Research Center, ADA Foundation, Gaithersburg, MD 20899, USA; stanislav.frukhtbeyn@nist.gov (S.A.F.); anthony.giuseppetti@nist.gov (A.A.G.); ugochukwu.okeke@nist.gov (U.C.O.); drago.skrtic@nist.gov (D.S.)

**Keywords:** dental materials, amorphous calcium phosphate, antimicrobial methacrylate monomers, antimicrobial and remineralizing composites

## Abstract

A trend for the next generation of polymeric dental restoratives is to incorporate multifunctional capabilities to regulate microbial growth and remineralize tooth surfaces. Polymerizable 2-(methacryloyloxy)-*N*-(2-(methacryloyloxy)ethyl)-*N*,*N*-dimethylethan-1-aminium bromide (IDMA1) and *N*,*N*′-([1,1′-biphenyl]-2,2′-diylbis(methylene))bis(2-(methacryloyloxy)-*N*,*N*-dimethylethan-1-aminium) bromide (IDMA2), intended for utilization in bi-functional antimicrobial and remineralizing composites, were synthesized, purified with an ethanol-diethyl ether-hexane solvent system, and validated by nuclear magnetic resonance (^1^H and ^13^C NMR) spectroscopy, mass spectrometry, and Fourier-transform infrared spectroscopy. When incorporated into light-curable urethane dimethacrylate (UDMA)/polyethylene glycol-extended UDMA (PEG-U)/ethyl 2-(hydroxymethyl)acrylate (EHMA) (assigned UPE) resins, IDMAs did not affect the overall resins’ hydrophilicity/hydrophobicity balance (water contact angle: 60.8–65.5°). The attained degrees of vinyl conversion (DVC) were consistently higher in both IDMA-containing copolymers and their amorphous calcium phosphate (ACP) composites (up to 5% and 20%, respectively) reaching 92.5% in IDMA2 formulations. Notably, these high DVCs values were attained without an excessive increase in polymerization stress. The observed reduction in biaxial flexure strength of UPE-IDMA ACP composites should not prevent further evaluation of these materials as multifunctional Class V restoratives. In direct contact with human gingival fibroblasts, at biologically relevant concentrations, IDMAs did not adversely affect cell viability or their metabolic activity. Ion release from the composites was indicative of their strong remineralization potential. The above, early-phase biocompatibility and physicochemical tests justify further evaluation of these experimental materials to identify formulation(s) suitable for clinical testing. Successful completion is expected to yield a new class of restoratives with well-controlled bio-function, which will physicochemically, mechanically, and biologically outperform the conventional Class V restoratives.

## 1. Introduction

Most contemporary dental restoratives recover a tooth’s anatomy and function, and yield good aesthetics [1,2]. These materials depend on adhesive systems of largely similar chemical composition, but with lower filler content as means of bonding to tooth structure. While concerns exist about their mechanical properties and abrasion resistance, their major disadvantage is the compromised bonding integrity that leads to the gaps at the adhesive/tooth interface, bacterial microleakage, and secondary caries. The bioactivity of the restoratives typically stems from the release of remineralizing ions (fluoride, calcium (Ca), and phosphate (PO_4_)), or their combination [3,4,5,6]. Our group has been on the forefront of the research on polymeric, amorphous calcium phosphate (ACP)-based materials for over two decades. Building on systematic structure-composition-property relationship studies [7,8], we have designed remineralizing polymeric composites that release Ca and PO_4_ ions in a sustainable manner and efficiently restore tooth mineral lost to decay [9,10]. While protecting from demineralization and regenerating mineral lost to caries, these restoratives do not guard against the ingression and accumulation of microorganisms at the restoration site. Therefore, recurrent dental caries remains a critical issue, even with the new(est) generation of dental restoratives. Adding antimicrobial (AM) functionality to these materials is seen as one of the enduring challenges, aimed at reducing the need for repetitive clinical intervention. Quaternary ammonium methacrylates, known for their AM action against both Gram-positive and Gram-negative bacteria, are typically utilized to impart AM activity to polymeric dental materials. They are, however, quite toxic due to the various biological effects of the quaternary ammonium “head” and its metabolism [11,12]. Multiple studies have indicated that quaternary ammonium compounds destroy cell membrane integrity and eventually lead to cell death [13,14,15]. Their AM action reportedly depends on the type of counter-ion [11], pendant active groups [16], molecular weight, and length of the alkyl chains [17]. So far, these structure/performance studies have not been successfully implemented to yield dental restorative(s) with sustained AM action [11,16,17,18,19,20]. Initially, most attention has been given to methacryloyloxydodecyl pyrimidinium bromide and its copolymer [1,21,22,23]. However, the composites with methacryloyloxydodecyl pyrimidinium bromide show poor color stability; thus, their use is limited to areas where aesthetics is not an issue. Among various quaternary ammonium compounds proposed for utility in bonding agents [24,25] and dental resin composites [26,27,28], ionic dimethacrylates were identified as viable candidates for offsetting concerns related to quaternary ammonium methacrylates. Dr. Xu’s group has been active in integrating quaternary ammonium dimethacrylates (QADMs) alone or in combination with nanosilver (nAg) and/or nano-ACP (nACP) into resins, based on conventional monomers (Table 1). While collectively these recent studies indicate the potential of using QADMs as AM agents in dental materials, there are still knowledge gaps and deficiencies regarding: (1) optimization of the resin composition; (2) purity and structural characterization of the synthesized agent; and (3) in vitro cytotoxicity evaluations with biologically relevant QADMs concentrations.

In this study, we synthesized, purified, and characterized 2-(methacryloyloxy)-*N*-(2-(methacryloyloxy)ethyl)-*N*,*N*-dimethylethan-1-aminium bromide (IDMA1) and *N*,*N*′-([1,1′-biphenyl]-2,2′-diylbis(methylene))bis(2-(methacryloyloxy)-*N*,*N*-dimethylethan-1-aminium) bromide (IDMA2). Next, we assessed in vitro cytotoxicity of IDMAs using a standard direct contact method. We incorporated IDMA1 or IDMA2 into light-curable urethane dimethacrylate (UDMA), polyethylene glycol-extended UDMA (PEG-U)/ethyl 2-(hydroxymethyl)acrylate (EHMA) resins (designated UPE resin) and assessed IDMAs effect on resins’ hydrophobic/hydrophilic balance. IDMA/UPE copolymers were assessed for degree of vinyl conversion (DVC) and biaxial flexural strength (BFS). Furthermore, we synthesized and validated ACP filler, homogenized it by milling and incorporated it into UDMA-based resins. The ensuing composites were evaluated for DVC, mechanical strength, and remineralizing potential. The underlying hypotheses were that: (1) IDMAs will have no detrimental effect on physicochemical and/or mechanical properties of copolymers; and (2) reduced DVC and/or mechanical stability of ACP/UPE-IDMA composites will be compensated by their remineralizing potential.

## 2. Results

### 2.1. IDMAs: Structural Validation

**Figure 1:**
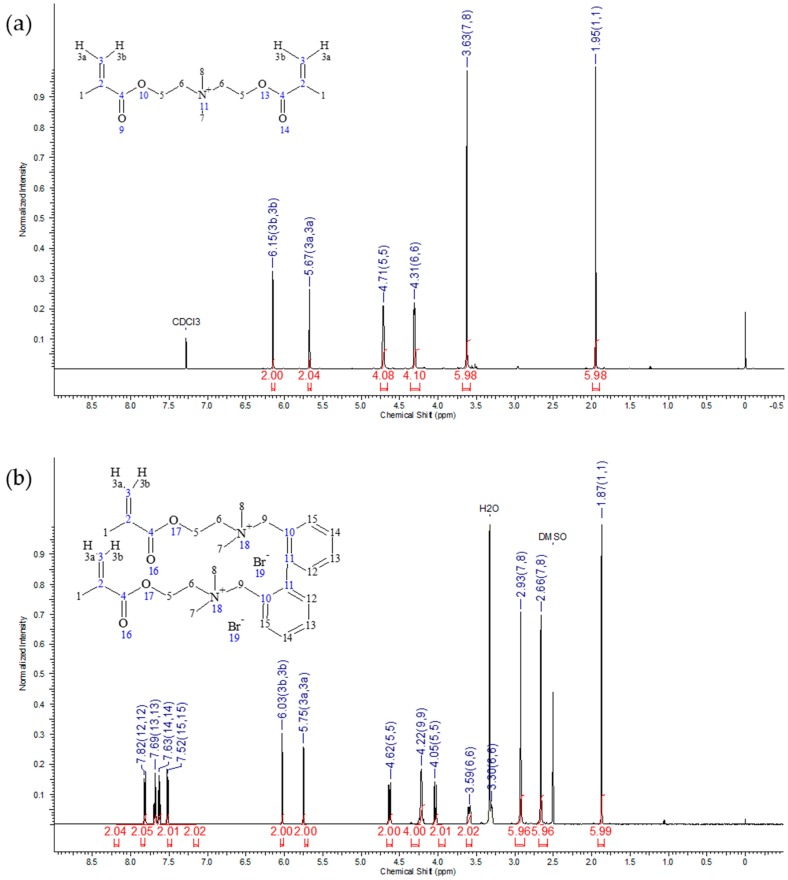
Typical ^1^H NMR spectrum of (**a**) IDMA1 and (**b**) of IDMA2.

**Figure 2:**
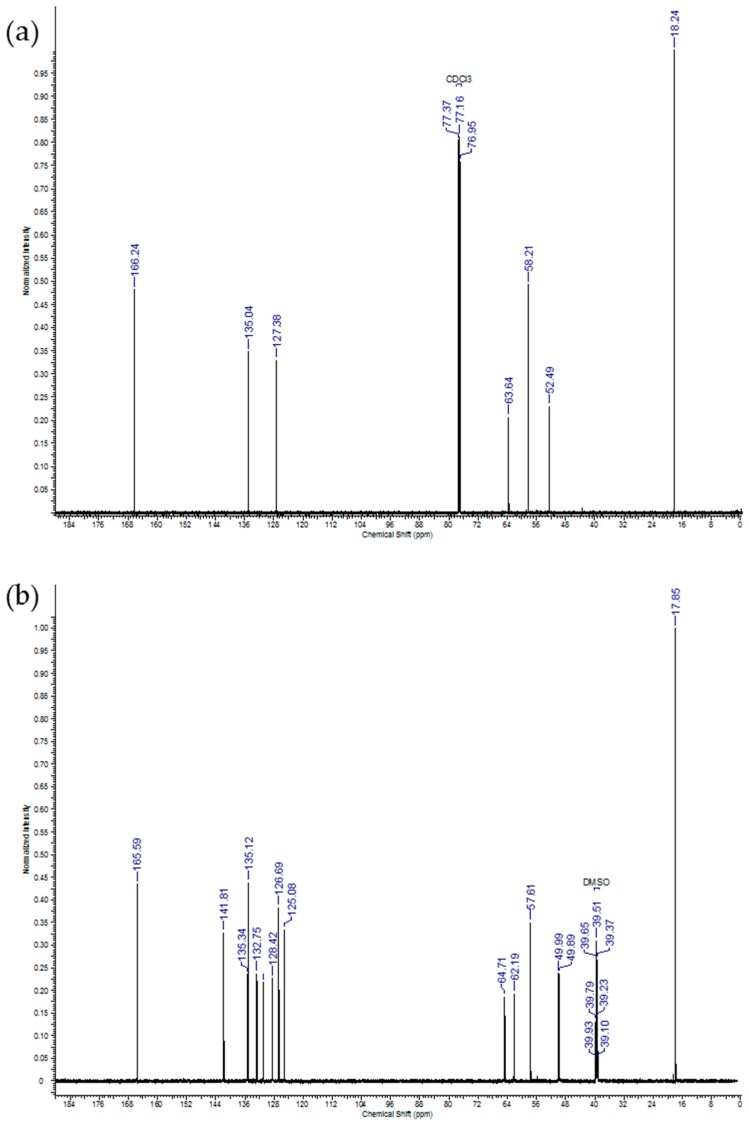
Typical ^13^C NMR spectrum of (**a**) IDMA1 and (**b**) of IDMA2.

Assigned structures of IDMAs were verified by nuclear magnetic resonance (NMR), mass spectrometry (MS), and Fourier-transform infrared spectroscopy (FTIR). ^1^H NMR and ^13^C NMR spectra of IDMA1 and IDMA2 are shown in Figure 1 and Figure 2, respectively with the accompanying assignments of chemical shifts to their structures (Table 2 and Table 3).

**Figure 3:**
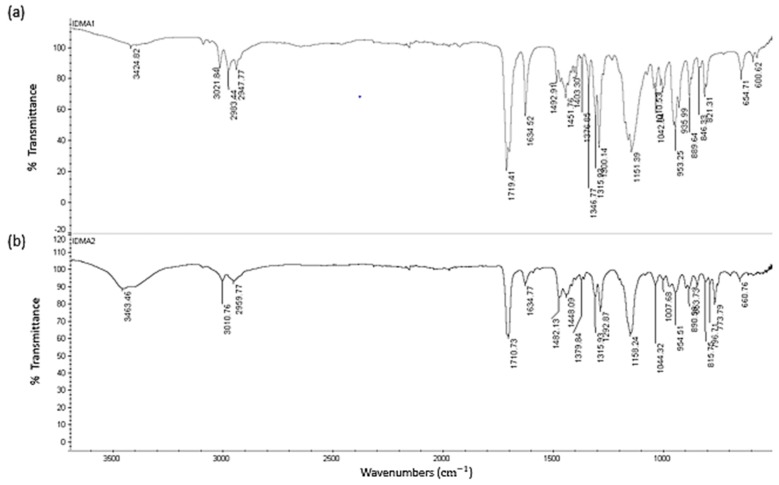
Typical FTIR spectrum of (**a**) IDMA1 and (**b**) of IDMA2.

FTIR spectra of IDMA1 and IDMA2 are shown in Figure 3. Both IDMA1 and IDMA2 are hygroscopic and gain moisture when stored in the open. Their hygroscopicity is verified by a broad peak at ~3400–3300 cm^−1^ in their FTIR spectra. Generally, the hygroscopic nature of quaternary ammonium salts have been demonstrated by X-ray crystallography and is attributed to hydrogen bonding between water molecules and quaternary ammonium’s bromide ion (Br⋯H–O–H) [30].

MS of IDMA1 (C_14_H_24_NO_4_Br) generated prominent peaks at *m*/*z* 270.35 (C_14_H_24_NO_4_^+^, calculated *m*/*z* 270.17) and at *m*/*z* 113.06 (C_6_H_9_O_2_^+^, calculated *m*/*z* 113.06). MS of IDMA2 (C_30_H_42_N_2_O_4_Br_2_) yielded prominent peaks at *m*/*z* 573.55/575.58, (C_30_H_42_N_2_O_4_Br^+^, calculated *m*/*z* 573.23/575.23); 416.33/418.29 (C_22_H_27_NO_2_Br^+^, calculated *m*/*z* 416.12/418.12); 179.18 (C_22_H_34_N_2_O_2_^+2^, calculated *m*/*z* 179.13); 113.07 (C_6_H_9_O_2_^+^, calculated *m*/*z* 113.07) (data not shown).

### 2.2. IDMAs: Biocompatibility

#### 2.2.1. Cellular Metabolic Activity

**Figure 4:**
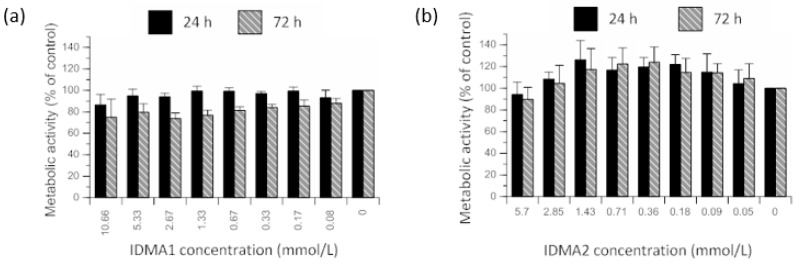
Percent control value of metabolic activity of HGF cells exposed for 24 h or 72 h to two-fold serial dilutions of (**a**) IDMA1 (≤10.66 mmol/L) or (**b**) IDMA2 (≤5.7 mmol/L). Data represent mean ± standard error of the mean (SEM) for five or more independent replicates tested in triplicate.

To determine the cytotoxic potential of QADMs targeted for dental restorations, human gingival fibroblasts (HGFs) were exposed to two-fold serial dilutions of IDMA1 (≤10.66 mmol/L). No statistical differences were observed when considering the main effect of IDMA1 concentration on the metabolic activity of HGFs (Figure 4a). However, time of IDMA1 exposure exerted a significant (*p* ≤ 0.05) main effect on cellular metabolic activity. When comparing the effect of time, without regard to concentration, the cellular metabolic activity was consistently lower (~13% difference of means) after 72 h of exposure.

IDMA2 concentration (≤5.7 mmol/L) did not exert a significant main effect on HGF metabolic activity (Figure 4b). Further, cellular metabolic activity was not statistically affected by the time of IDMA2 exposure (up to 72 h).

For the testing of both IDMAs, negligible optical density values were observed in control wells, containing no cells. Controls (with or without cells) in which the tetrazolium compound was omitted resulted in low optical densities. Positive controls, containing non-exposed cells were not significantly different from cells that were previously stained with ethidium homodimer-1 and calcein (data not shown).

#### 2.2.2. Cellular Viability

**Figure 5:**
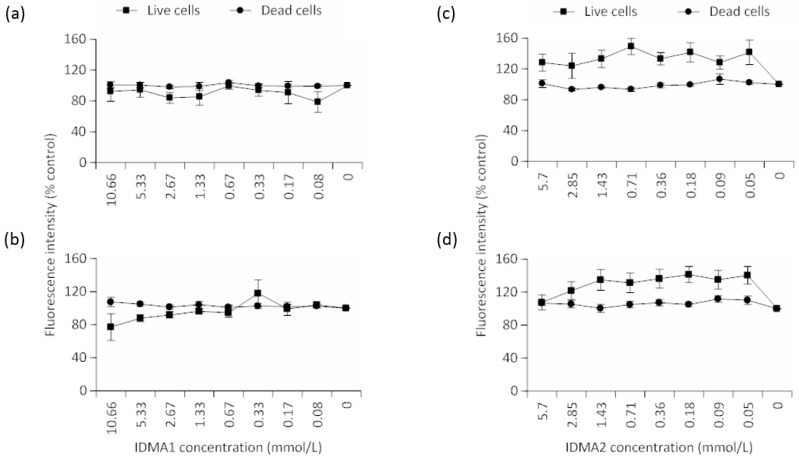
Percent control value of viability of HGF cells exposed to two-fold serial dilutions of IDMA1 (≤10.66 mmol/L) for (**a**) 24 h or (**b**) 72 h to two-fold serial dilutions of IDMA1. Percent control value of viability of HGF cells exposed for (**c**) 24 h or (**d**) 72 h to two-fold serial dilutions of IDMA2 (≤5.70 mmol/L). Data represent mean ± SEM for five independent replicates tested in triplicate.

As with cell metabolic activity profiles, viability data indicated an overall insignificant effect of IDMA1 concentration (Figure 5a,b). Cell viability was 5.5% lower (difference of means without regard to concentration) between the time points. Although this comparison was insignificant, a trend for decreased viability was observed at higher concentrations after 72 h exposure.

When comparing IDMA2 concentrations within a given time point, no statistical differences were observed when the number of viable cells were compared (Figure 5c,d). Likewise, IDMA2 concentrations did not significantly affect the number of dead cells. A statistical main effect (*p* ≤ 0.05) of time was observed in the number of dead cells (i.e., nearly a 7% increase in the difference of means).

### 2.3. ACP Filler Remineralizing Ability

**Figure 6:**
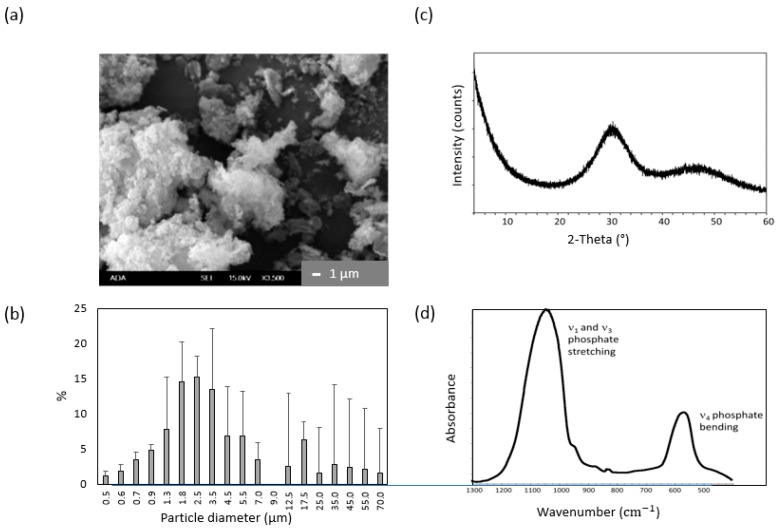
Validation of synthesized ACP. (**a**) Morphology of ACP particles under scanning electron microscopy (×3500 magnification); (**b**) Differential particle size distribution (mean values ± SD, *n* = 2); (**c**) XRD pattern; (**d**) FTIR spectrum of milled ACP utilized to fabricate ACP/UPE-IDMA composites.

Milled ACP utilized to fabricate ACP/UPE-IDMA composites had a median dimeter (d_m_) of 2.73 ± 0.17 µm, which represents a 45% reduction compared to as-made ACP (d_m_ = 4.93 ± 0.52 µm). The particle size distribution (PSD) range (submicron to 70 μm) apparently was not affected by milling (data not shown). X-ray diffraction (XRD) spectrum of ACP showed two diffuse broad bands ((24–36) and (40–55) 2θ degrees); patterns typical for calcium phosphates with low crystallinity. Corresponding FTIR spectra exhibited two wide bands typical for PO_4_ stretching and PO_4_ bending in the region of 1200–900 cm^−1^ and 630–500 cm^−1^, respectively. Typical morphology (scanning electron microscopy data), results of PSD analysis, and structural verification by FTIR and XRD are presented in Figure 6.

**Figure 7:**
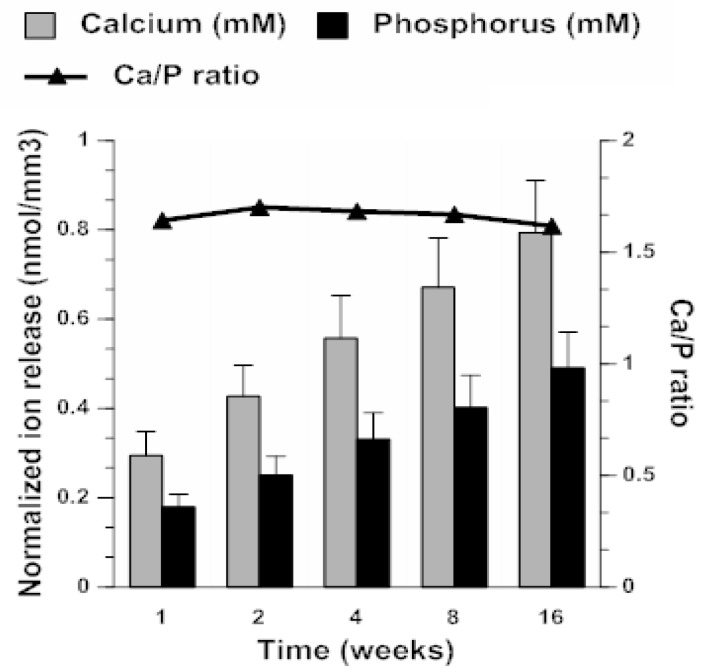
Kinetics of ion release from ACP UPE composites upon prolonged aqueous immersion. Indicated are mean values ± SD of triplicate measurements.

Kinetic profiles of Ca and phosphorus (P) release from milled-ACP UPE composites (Figure 7) revealed that within 4 months of aqueous immersion, a plateau (maximum) ion concentration had not been reached. The supersaturation conditions (SR >> 1) attained at any given time point were notably above the minimum necessary for the apatite re-deposition. It is particularly significant that the attained solution Ca/P ratio (average 1.66 ± 0.03) is practically identical to the apatite’s theoretical Ca/P value of 1.66, meaning that solution conditions created by composite’s immersion are highly conducive to apatite formation.

### 2.4. Preliminary Physicochemical Evaluation of Resins and Composites

**Figure 8:**
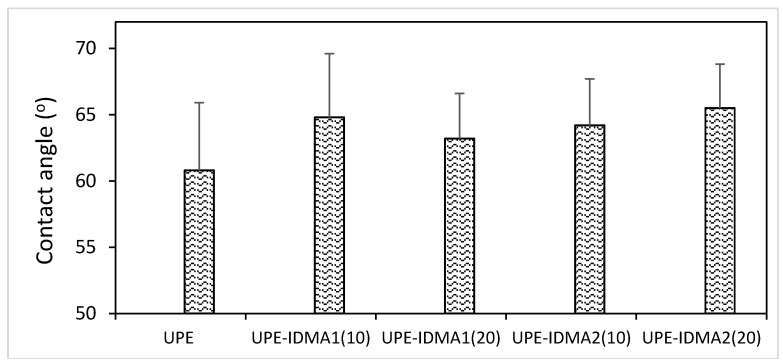
Changes in the overall hydrophilicity/hydrophobicity of the resins upon introduction of IDMAs (at 10 and 20 mass %) into UPE formulation. Indicated values represent mean ± SD for four replicate measurements.

Hydrophobic/hydrophilic balance of UPE resins (average contact angle 60.8 ± 5.1°) was not affected significantly by either the type or the concentration of IDMA monomer included in the matrix (average contact angle 64.4 ± 5.1°) (Figure 8).

**Figure 9:**
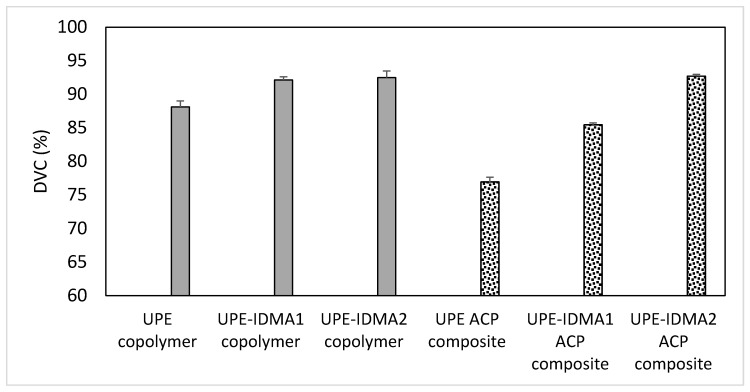
DVC attained in copolymers (near infra-red measurements; 24 h post cure) and the corresponding ACP composites; 15 min post-cure (curing conditions: 40 s, 500 mW/cm^2^). Shown are mean values ± SD for three replicate measurements.

**Figure 10:**
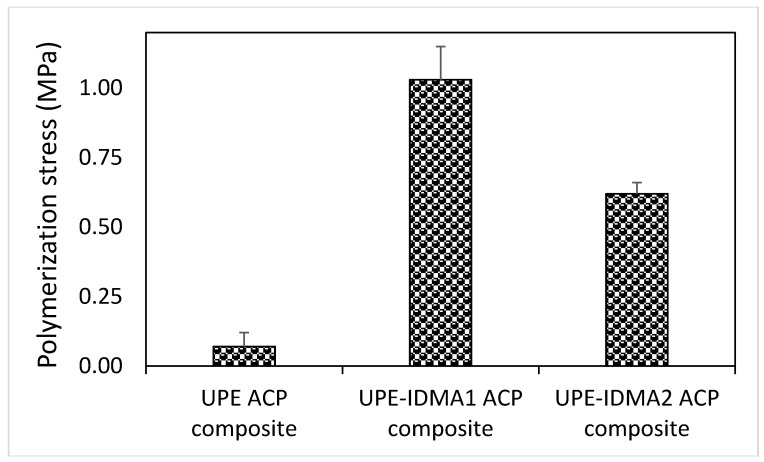
Polymerization stress (mean values ± SD for three replicate measurements) developed in ACP composite specimens 15 min after photo irradiation.

DVC results (Figure 9) revealed that in both copolymer and composite series, significant differences (*p* ≤ 0.001) existed between no IDMA, IDMA1, and IDMA2 formulations. The post-hoc multiple pair comparisons indicated that the observed up to 5% and 20% (copolymer and composite series, respectively) increases in DVC of IDMA-containing formulations are significant (*p* ≤ 0.001). It is noteworthy that the high DVC values (85.5–92.7%) attained in the experimental ACP UPE-IDMA composites were, generally, not accompanied with the excessive polymerization stress (Figure 10). Notably, in IDMA2 composites that reached the highest level of vinyl conversion, the measured polymerization stress was almost 40% lower than in IDMA1 composites.

**Figure 11:**
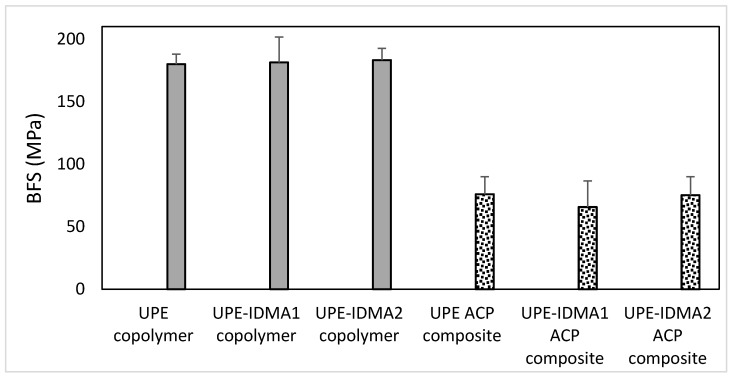
BFS of copolymers and composites. Indicated are mean values ± SD for *n* = 5 (copolymer group) and *n* = 4 (composite group).

The results of biaxial flexural strength (BFS) testing of copolymer and composite specimens are summarized in Figure 11. Introducing IDMA monomers did not affect the BFS of copolymers (average 181.5 ± 12.6 MPa). Incorporation of ACP filler, however, resulted in 60% reduction in the BFS of composites (on average 72.2 ± 16.5 MPa).

## 3. Discussion

Any AM agent’s biocompatibility is likely to be compromised by the presence of significant amounts of impurities, unreacted reagents, and/or residual solvents in the material. Therefore, the ability to purify crude IDMAs with an ethanol-diethyl ether-hexane solvent system, developed as a part of this study, is essential to minimize the potential toxicity of an AM agent towards host cells. The synthetic/purification approach that has yielded high purity (≥97%) IDMAs has strong potential to be extended to design a new generation of monomers with improved resin miscibility (AM_misc_), adhesiveness to teeth (AM_adh_) and coupling with the remineralizing ACP filler (AM_cpl_). A successful fabrication of high-purity AM_misc_, AM_adh_, and AM_cpl_ monomers, anticipated in near future, will enable us to clearly delineate the ability of these new agents to cross-link with UDMA-based resins, while safeguarding their AM function and maintaining satisfactory mechanical performance of antimicrobial and remineralizing (AMRE) composites.

Although AM QADMs have received considerable attention for use in dental restoratives, the reports on their biocompatibility are generally scarce. Biocompatibility screenings of AM agents are commonly performed in direct contact with polymer, rather than by exposing cells to AM concentrations corresponding to their predicted levels in the oral milieu. The first published IDMA1 cytotoxicity study involved a conventional polymer matrix and mouse macrophage-like cells (RAW 264.7) [26]. Polymers containing 10 mass % IDMA1 reduced cell density without affecting their viability. At IDMA contents >10 mass %, both cell density and viability were reduced. Evaluation of the residual cells, remaining after polymer disk removal, led to the conclusion that leachables did not affect RAW 264.7 cells. Another study employing HGFs [24] found the eluent toxicity of bonding agent containing QADM equaled no-QADM controls. In both of the above studies, the leached extract was not characterized and QADM (IDMA1) concentrations were not determined. Our group considered the in vitro cytotoxicity of an AM agent, at biologically pertinent levels and in direct contact with relevant cells, as the crucial prognosticator for material(s) development and justification for further exploration. We have initially evaluated IDMA1 and IDMA2 employing mouse subcutaneous connective tissue fibroblasts [31] and found no adverse effect on cell viability or their metabolic activity at biologically relevant concentrations. In this study, we report no toxicity at biologically relevant concentrations of both IDMA1 and IDMA2 towards HGFs. The tested concentrations of IDMA1 and IDMA2 were calculated based on the accelerated leachability data reported for UDMA/PEG-U/2-hydroxyethyl methacrylate (HEMA) copolymers and their ACP composites [8]. It is reasonable to expect that, due to the high DVCs attained in our experimental formulations, the concentration of IDMA1 or IDMA2 leaching from the cured materials will not exceed leachability of small molecular weight and highly diffusive HEMA from UDMA/PEG-U/HEMA formulations. A quantitative leachability study of UPE-IDMA resins and composites will be executed in the near future to confirm our hypothesis. In a later phase of AMRE composite development, the bio-evaluation tests will be expanded to include genotoxicity, immunotoxicity, and inflammation studies.

UPE-IDMA copolymers attained DVCs (92.1–92.5%) that significantly exceeded DVC values of the 2,2-bis[p-(2-hydroxy-3-methacryloxypropoxy)phenyl]propane (Bis-GMA)**/**triethylene glycol dimethacrylate (TEGDMA)/IDMA counterparts (67.9–70.7%) [26]. Such high DVC values are likely due to a delayed vitrification point incited by the inclusion of PEG-U, EHMA, and IDMA into resin and the resulting reduction in the fraction of the base monomer (UDMA) in the formulation. These DVCs suggest that chain mobility of a cross-linked polymer is small and that the pathways for unreacted monomers to leach-out are limited. These experimental findings are in line with the results of our earlier structure/composition property studies indicating the improved DVCs were achieved by substituting a more flexible aliphatic UDMA/PEG-U mixture for rigid, aromatic base monomer Bis-GMA and replacing TEGDMA with diluent monomers, such as HEMA or EHMA [32,33,34]. It is particularly important that the elevated DVC in these systems was not accompanied with excessive polymerization stress. Moreover, in UPE-IDMA2 ACP composites, compared to the IDMA1 counterparts, higher DVC were attained while generating less stress. This phenomenon could be explained by the fact that during the curing process, not all the polymerization shrinkage is transformed into stress but is rather compensated by polymer reorganization. Internal rotation around the central biphenyl bond [35,36] of IDMA2 may allow it to assume multiple conformations, ranging from a planar to a “twisted” form, which may be conducive to such polymer rearrangement to dissipate some of the polymerization-generated stress. It is noteworthy that stresses generated in UPE-IDMA ACP composites (0.6–1.0 MPa), while exceeding the values seen in no-IDMA UPE ACP counterparts, remained well below the polymerization stress levels reported for UDMA/PEG-U/HEMA ACP composites (3.3–4.5 MPa) [37].

Results of UPE-IDMA1 and UPE-IDMA2 resin wettability testing (average contact angle 64.4**°**) correlated well with the upper thresholds of contact angles reported for commercial resin-based composites (range 31.5–64.5**°**) [38]. High contact angle values generally imply lower water sorption associated with lesser tooth staining, plaque accumulation, and pathogen adhesion and proliferation. Since, collectively, these factors lower the risk of secondary caries development [39], lesser risk should be associated with the use of restoratives based on the proposed formulations.

Typically, in composites utilizing the reinforcing fillers, a positive correlation exists between the high DVCs and mechanical/chemical stability and the composites longevity [40]. This relationship does not, however, apply to UPE-IDMA ACP composites for a simple reason—the remineralizing ACP filler has no reinforcing ability. The observed reduction in BFS in going from copolymers to ACP composites (60.0% in this study) appears to be marginally lower than the reduction in BFS reported for ACP filled Bis-GMA-based (63.2% reduction) [41], ACP filled EBPADMA-based (69.5% reduction) [41] and ACP filled UDMA-based (69.9% reduction) [7] composites compared to the corresponding copolymers. These consistently lower BFS values seen in ACP composites, regardless of the actual resin matrix, suggest that ACP/resin interlocking is inadequate to achieve better mechanical properties. Consequently, ACP composites’ restorative potential should be restricted to dental applications where high mechanical strength is not the main requirement. Class V restorations, for which our experimental material is ultimately intended for, are an example of such applications. Therefore, the observed reduction in BFS of composites vs. copolymers in our experimental systems should not prevent further exploration of UPE-IDMA resins for intended AMRE application. This unwelcomed effect could possibly be ameliorated by introducing, in addition to the remineralizing ACP filler, reinforcing zirconia and silica particles, and/or mixture of silica plus barium silicate glass during AMRE composite fabrication.

The ion release data, recalculated in terms of solution supersaturation with respect to apatite and/or enamel, confirmed the potential of UPE-IDMA ACP composites to regenerate tooth mineral. This finding supports our previous findings [7] that the compositional changes in the resin phase of ACP bioactive composites do not compromise their remineralizing ability. Efficacy of ACP remineralizing composites has so far been documented in two in vitro studies involving bovine [9] and human teeth [10]. To overcome a significant gap that typically exists between the in vitro and in vivo evaluations, testing of the prototype AMRE materials should involve their testing in preclinical settings.

In conclusion, IDMAs of enhanced purity exhibited minimal or no cytotoxicity towards host cells. Their incorporation into UDMA-based matrices did not alter the resins’ hydrophobic/hydrophilic balance while improving degree of vinyl conversion and even, in IDMA2 systems, reducing polymerization-generated stress. The ensuing ACP/UDMA-based composites displayed strong remineralizing potential. The observed reduction in BFS of ACP composites should not compromise their intended utilization as AMRE restoratives for Class V caries. The preliminary biological and physicochemical assessments of UDMA/PEG-U/EHMA copolymers and their ACP composites validate their further exploration.

## 4. Materials and Methods

### 4.1. Materials

IDMAs: 2-(*N*,*N*-dimethylamino)ethyl methacrylate (DMAEMA), 2-bromoethyl methacrylate (BEMA), and 2,2′-bis(bromomethyl)-1,1′-biphenyl (bBrMbP), polymerization inhibitor butylated hydroxytoluene, and the solvents (absolute ethanol, chloroform (CHCl_3_), deuterated chloroform (CDCl_3_), deuterated dimethyl sulfoxide (DMSO-*d*_6_), hexane, and diethyl ether) used for IDMA syntheses and their purification were purchased from Sigma-Aldrich Co., St. Louis, MO, USA.

Biocompatibility: Immortalized human gingival fibroblasts (HGF) and PriGrow III medium were purchased from Applied Biological Materials, Inc. (Richmond, BC, Cananda). Fetal bovine serum was obtained from American Type Culture Collection (Manassas, VA, USA). The LIVE/DEAD^®^ Viability/Cytotoxicity Kit and Dulbecco’s phosphate-buffered saline were acquired from Life Technologies, Corp., Grand Island, NY, USA. Methanol (meeting ACS specifications) was purchased from Sigma-Aldrich Co. The CellTiter 96^®^ AQueous One Solution Cell Proliferation Assay was obtained from Promega (Madison, WI, USA).

Resins: UDMA and PEG-U were donated by Esstech, Essington, PA. EHMA, camphorquinone (CQ), and ethyl-4-*N*,*N*-dimethylamino benzoate (4EDMAB) were purchased from Sigma-Aldrich Co.

ACP: Ca(NO_3_)_2_, Na_2_HPO_4_, Na_2_P_2_O_7_ and ZrOCl_2_ (ACP synthesis), 1-propanol (composite preparations), and NaCl, NaOH, and 4-(2-hydroxyethyl)piperazine-1-ethanesulfonic acid (HEPES) (ion release experiments) were acquired from Sigma-Aldrich Co.

All reagents/solvents were used as received.

### 4.2. IDMAs: Syntheses and Purification

For IDMA1 synthesis, DMAEMA (10 mmol), BEMA (10 mmol), BHT (1 mmol), and CHCl_3_ were mixed in a reaction vessel equipped with a reflux column. Reaction mixture was heated (55–60 °C) for 24 h and then washed with hexane. IDMA2 was synthesized by reacting DMAEMA (10 mmol) and bBrMbP (5 mmol) under conditions similar to those employed in IDMA1 synthesis. Crude IDMAs were purified by a multi-step process, using an ethanol-diethyl ether-hexane solvent system. Purified IDMA2 was freeze-dried overnight and the remaining impurities were washed away with CHCl_3_.

The yield of purified product of IDMA1 and IDMA2 was 25.6% and 79.5%, respectively of their theoretic value.

### 4.3. IDMAs: Structural Verification

The anticipated IDMAs’ structures were confirmed by ^1^H and ^13^C NMR, and MS. NMR spectral data (^1^H, ^13^C NMR, and HSQC) were interpreted to assign the C and H chemical shifts to their structure.

NMR: Spectra were obtained using a Bruker Avance 2 (600 MHz) spectrometer equipped with a Broadband Observe room temperature probe. Samples were run using CDCl_3_ and DMSO as solvents and tetramethylsilane (TMS) as an internal standard. Concentrations of the samples analyzed varied, but were generally within 0.1–0.5 M. HSQC was employed for spectra signal assignation.

MS: Mass spectroscope (Quattro Micro, Waters Corp., Millford, MA, USA) was operated in the positive ionization mode. Three kV potential was applied to the electrospray ionization needle and the de-solvation temperature was set at 250 °C. Nitrogen flow and sample injection rates were 500 L/h and 10 µL/min, respectively.

FTIR spectroscopy: FTIR spectra of IDMAs were recorded using a deuterated triglycine sulfate room temperature detector in the spectral range of 4000–500 cm^−1^ (Nexus 670 ThermoNicolet, Nicolet Instrument Corporation, Madison, WI, USA). The samples were measured on a diamond attenuated total reflection cell.

### 4.4. Biocompatibility Tests

Cell maintenance: Cells were maintained in PriGrow III Medium supplemented with 10% fetal bovine serum (37 °C, 5% CO_2_ environment). To initiate experiments, cells were obtained from a subconfluent culture. An improved Neubauer hemocytometer using trypan blue staining and bright field microscopy was utilized to determine cell viability. A Falcon™ 96-well, black/clear, flat-bottomed, tissue culture treated plate (Corning, Inc., Big Flats, NY, USA) were seeded with 10,000 viable cells/well.

Leachability constraints: The cells were exposed to two-fold serial dilutions of IDMAs after 48 h incubation. Concentrations (≤10.66 mmol/L for IDMA1 or ≤5.70 mmol/L for IDMA2) were used to approximate the maximum possible exposure, assuming 7% mass fraction in the composite with ≤2% leaching. An accelerated ACP UDMA/PEG-U/HEMA leachability study [8] was used to establish these experimental values. Negative control wells were without the dental monomers and/or cells. Cultures were assessed for viability and metabolic activity after 24 h and 72 h exposure. Experiments were performed on five independent occasions with triplicate wells on each plate.

Cell viability: After monomer exposure, cells were washed with Dulbecco’s phosphate-buffered saline (without calcium chloride and magnesium chloride). Cells were then incubated for 20 min at room temperature with ethidium homodimer-1 (1 µM) and calcein acetoxymethyl ester (0.658 μM) (optimal stain concentrations were pre-determined by serial dilution). Fluorescence of ethidium homodimer-1 (excitation 530 nm, emission 645 nm) and calcein (excitation 485 nm, emission 530 nm) was assessed with a Spectra Max M5 plate reader (Molecular Devices, Corp., Sunnyvale, CA, USA). Assay controls included live and 70% methanol-treated cells that were subjected to ethidium homodimer-1 and/or calcein acetoxymethyl ester. These control values were used to estimate the percentage of live and dead cells, according to the manufacturer’s instructions. In these calculations, the value associated with the live cells stained with calcein acetoxymethyl ester becomes mathematically influential. Consequently, inter-assay control limits were set for these wells (value of each triplicate well needed to be within 20% of their mean).

Cellular metabolic activity: The CellTiter 96^®^ AQueous One Solution Cell Proliferation Assay is a colorimetric method based on the reduction of tetrazolium salt by metabolically intact cells to produce a formazan product. As with other tetrazolium salt assays, it is believed that most of this reduction occurs outside the mitochondrial inner membrane [42].

After assessing cellular viability, the metabolic activity of the cells was estimated using the CellTiter 96^®^ AQueous One Solution Reagent. Specifically, the supernatant was removed and each well received 200 µL culture medium and 40 µL CellTiter 96^®^ AQueous One Solution Reagent. Subsequently, plates were incubated at 37 °C in a 5% CO_2_ environment for 40 min. An Epoch microplate spectrophotometer (BioTek Instruments, Inc., Winooski, VT, USA) set at 490 nm was used to determine optical densities. The optical densities were transformed into the percent increase or decrease of that observed in the control group (no monomer) to normalize assay responses. Wells that only contained an equal volume of culture medium served as controls.

### 4.5. Resin Formulation and Evaluation

UPE resins were formulated from the commercially available UDMA (50.91 mass %), PEG-U (18.18 mass %), EHMA (30.91 mass %) and a conventional visible light initiator system employing CQ (0.2 mass %) and 4EDMAB (0.8 mass %) [8]. Chosen 2.8:1.0:1.7 UDMA:PEG-U:EHMA mass ratio represents the average for the UDMA-based ternary formulations so far explored by our research team. IDMAs were blended into UDMA-based resins at 10 or 20 wt %. These concentrations were chosen based on the previously reported findings [26] that 10 wt % IDMA1 in Bis-GMA/TEGDMA polymers significantly reduced bacterial attachment while 30 wt % IDMA1 did not further reduce bacterial coverage, while diminishing macrophage density and activity. After combining the monomers, their mixture was magnetically stirred (38 rad/s) at room temperature until achieving uniform consistency.

Homogenized resin was pipetted into stainless steel molds (for contact angle evaluation; 13 mm diameter, 1.4 mm height) or hollow glass beams (DVC: 1.9 × 1.9 × 25 mm). Each side of the mold was covered sequentially with Mylar film and a glass slide and the assembly was clamped. Copolymerization was initiated by irradiating specimens for 120 s on each side (Triad 2000, Dentsply International, York, PA, USA). After curing, copolymer samples were de-molded and kept in the dark, under vacuum, for ≥24 h.

Changes in water contact angle (sessile drop technique), an indication of resin’s hydrophobicity/hydrophilicity upon introduction of IDMAs were measured by a drop shape analyzer DSA-100 (Krüss, Matthews, NC, USA; *n* = 4/experimental group).

DVC of UPE-IDMA copolymer specimens was determined by NIR spectroscopic method that monitors the reduction in C=C absorption at 6165 cm^−1^ in the overtone region. By maintaining a constant specimen thickness, a need to use an invariant absorption band as an internal standard has been circumvented. The NIR spectra (*n* = 3/group) were collected before cure and 24 h post-cure. The DVC was calculated according to the following formula:

DVC (%) = (1 − Area_polymer_/Area_monomer_) × 100 (1)

where Area_polymer_ and Area_monomer_ represent the areas of 6165 cm^−1^ = C–H bond after and before curing, respectively.

The BFS of copolymer specimens (*n* = 5/group) was determined using a piston-on-three-ball loading cell with computer-controlled Universal Testing Machine (Instron 5500R; Instron Corp., Canton, MA, USA) supported by Testworks4 software. The BFS values were calculated according to the ASTM F394-78 specification. The same methodology was applied to measure BFS of ACP composite specimens.

### 4.6. Remineralizing ACP Composites

#### 4.6.1. ACP Filler: Synthesis and Characterization

ACP was synthesized employing our well-established protocols [32,33,34]. It precipitated instantaneously in a closed system at 22 °C upon rapidly mixing equal volumes of an 800 mmol/L Ca(NO_3_)_2_ solution and a 536 mmol/L Na_2_HPO_4_ solution that contained a molar fraction of 2% Na_4_P_2_O_7_ (to prevent premature ACP conversion to hydroxyapatite). The suspension was filtered, the solid phase washed subsequently with ice-cold ammoniated water and acetone, and then lyophilized. Amorphousness of the filler was validated by FTIR and X-ray diffraction (XRD; Rigaku X-ray diffractometer, Rigaku/USA Inc., Danvers, MA, USA). Particle size distribution (PSD; SA-CP3 analyzer, Shimadzu Scientific Instruments Inc., Columbia, MD, USA) was assessed before (as-made ACP) and after milling (5 g of ACP in 15 g of 1-propanol; 2 h at 200 rpm, Pulverisette 7 ball mill (Fritsch, Germany). To avoid exposure to humidity, ACP was kept under vacuum (2.7 kPa) until used in composite preparation.

#### 4.6.2. Fabrication of ACP Composites

ACP was dispersed in 1-propanol, sonicated 1 h in a water bath, and mixed with the appropriate amount of UPE or UPE-IDMA resin to yield composite of homogeneous appearance containing 60 mass % resin and 40 mass % filler. Solvent was evaporated at 22 °C, under vacuum (overnight) and continuous magnetic stirring. The composite paste was then tightly packed into stainless steel molds (13 mm diameter, 1.4 mm height). Both sides of the mold were covered sequentially with Mylar film and a glass slide. The assembly was clamped and each side irradiated for 120 s with visible light (Triad 2000, Dentsply International, York, PA, USA). After curing, samples were de-molded and kept in the dark, under vacuum, for ≥24 h before testing. More detailed descriptions of composite fabrication have been reported [32,33,34].

#### 4.6.3. Ion Release as Predictor of Remineralization Potential of ACP Composites

The ion release experiments were based on thermodynamic tenets, which have been previously used to indicate a composites potential for mineral repair [9,10]. For experiments (*n* = 5/group), composite disk specimens were individually immersed in 90 mL of HEPES-buffered (7.4 pH) 0.13 M NaCl solution. Immersing solutions were replaced with fresh buffers at 1, 2, 4, 8, and 16 weeks. Solution Ca and P concentrations were determined by atomic emission spectroscopy (AES; Prodigy High Dispersion ICP, Teledyne Leeman Labs, Hudson, NH, USA). The AES data were used to calculate the solutions’ thermodynamic stability (Chemist Application version 1.0.1.0; Micromath Research, St. Louis, MO) and their supersaturation (supersaturation index, that relates the ion activity product defined as [Ca]^10^[PO_4_]^6^[OH]^2^ and hydroxyapatite’s or enamel’s solubility product) [41].

#### 4.6.4. Simultaneous Assessment of Polymerization Stress and DVC

Simultaneous real-time measurements of polymerization stress and DVC of UPE-IDMA ACP composites employed the cantilever beam-based tensometer coupled with an in situ NIR spectrometer [43]. The synchronized stress/DVC data were collected for 15 min at clinically-relevant instrumental compliance of 0.33 µm/N. The 2.5 mm sample diameter and 2 mm sample height corresponded to a C-factor (ratio of bonded/unbonded area) of 0.625 [44,45]. Specimens, placed between two flat methacrylate-silane treated quartz rods (to promote specimen/rod adhesion), were cured (LZ1-10DB0, Mouser Electronics, Mansfield, TX, USA) for 40 s at 500 mW/cm^2^ light intensity. The axial stress due to the specimen’s shrinkage upon polymerization caused a deflection of the beam. This deflection, recorded by a displacement sensor, was used to quantify the polymerization stress. To determine the DVC (Equation (1)), NIR signal was guided through the sides of the specimen via optical fiber cable. 

### 4.7. Statistical Analyses

Analysis of variance (ANOVA) was used to analyze experimental data as a function of material makeup, storage/exposure time or any other relevant factor included in the experimental design. When overall statistically significant effects were found with ANOVA, multiple comparisons (2-sided, α = 0.05) were used to determine significant differences between groups (SigmaPlot™ 11.0; Systat^®^ Software, Inc., San Jose, CA, USA or Excel2016, Microsoft Corp. (Redmond, WA, USA). Excel2016 or DeltaGraph6 for Windows^®^ (Red Rock Software, Inc., Salt Lake City, UT, USA) were used to create graphics.

## Figures and Tables

**Table 1:** Antimicrobial efficacy expressed as n-fold decrease in biofilm formation of experimental QADM-based dental materials.

Ref.	Dental Material	Experimental Groups	Fold Decrease ^1^
[28]	**Composite** BisGMA-TEGDMA	Control: Commercial composite	
		20% nACP + 6% QADM (aged 1 day)	1.36
		20% nACP + 0.1% nAg (aged 1 day)	1.75
		20% nACP + 6% QADM + 0.1% nAg (aged 1 day)	2.69
[29]	**Composite** BisGMA-TEGDMA	Control: Composite (no fluoride)	
		19.5% nACP	1.25
		19.5% nACP + 7% QADM	1.73
		19.5% nACP + 0.028% nAg	1.98
		19.5% nACP + 7% QADM + 0.028% nAg	3.00
[24]	**Primer/adhesive** Primer: HEMA-acrylic/itaconic acids Adhesive: BisGMA-HEMA	Primer control + Adhesive control	
		Primer w/10% QADM + Adhesive w/10% QADM	1.65
		Primer w/0.05% nAg + Adhesive w/0.05% nAg	2.38
[25]	**Primer/adhesive** Primer: PMGDM-HEMA Adhesive: BisGMA-TEGDMA	Primer control + Adhesive control	
		Primer w/0.1% nAg + Adhesive control	2.70
		Primer w/0.1% nAg + Adhesive w/0.1% nAg, 10% QADM	2.86
		Primer w/0.1% nAg + Adhesive w/0.1% nAg, 10% QADM, 10% nACP	3.07
		Primer w/0.1% nAg + Adhesive w/0.1% nAg, 10% QADM, 20% nACP	2.98
		Primer w/0.1% nAg + Adhesive w/0.1% nAg, 10% QADM, 30% nACP	4.57
		Primer w/0.1% nAg + Adhesive w/0.1% nAg, 10% QADM, 40% nACP	5.11

^1^ indicates n-fold decrease in metabolic activity (mean absorbance of control/mean absorbance of experimental group) of bacterial biofilms. Mean absorbance data extracted from published figures.

**Table 2:** Assignments of ^13^C and ^1^H NMR chemical shifts of IDMA1.

Atom #	^13^C Chemical Shift, ppm	^1^H Chemical Shift, ppm	# H`s	Signal Splitting
1	18.2	1.95	6	singlet
2	135.0		0	
3	127.4	5.67, 6.15	2, 2	singlets
4	166.2		0	
5	58.2	4.71	4	multiplet
6	63.6	4.31	4	multiplet
7, 8	52.5	3.63	6	singlet

**Table 3:** Assignments of ^13^C and ^1^H NMR chemical shifts of IDMA2.

Atom #	^13^C Chemical Shift, ppm	^1^H Chemical Shift, ppm	# H`s	Signal Splitting
1	17.9	1.87	6	singlet
2	135.3		0	
3	126.7	5.75, 6.03	2, 2	singlets
4	165.6		0	
5	64.7	4.04, 4.63	2, 2	doublets
6	62.2	3.30, 3.59	2, 2	multiplets
7, 8	49.9, 50.0	2.66, 2.93	6, 6	singlets
9	57.6	4.22	4	broad
10	141.8		0	
11	125.1		0	
12	135.1	7.82	2	multiplet
13	131.0	7.69	2	triplet
14	128.4	7.63	2	triplet
15	132.8	7.52	2	multiplet

## Abbreviations

List of Acronyms	
ACP	amorphous calcium phosphate
ADA	American Dental Association
AM	antimicrobial
AM_adh_	antimicrobial monomer with improved adhesiveness
AM_cpl_	antimicrobial monomer with improved coupling to filler
AM_misc_	antimicrobial monomer with improved miscibility
AMRE	antimicrobial and remineralizing
ANOVA	analysis of variance
BEMA	2-bromoethyl methacrylate
BFS	biaxial flexural strength
Bis-GMA	2,2-bis[p-(2-hydroxy-3-methacryloxypropoxy)phenyl]propane
Ca	calcium
CQ	camphorquinone
d_m_	median diameter
DVC	degree of vinyl conversion
4EDMAB	ethyl-4-*N*,*N*-dimethylamino benzoate
EHMA	ethyl 2-(hydroxymethyl)acrylate
FTIR	Fourier-transform infrared spectroscopy
HEMA	2-hydroxyethyl methacrylate
HGF	human gingival fibroblast
HSQC	heteronuclear single quantum coherence
IDMA1	2-(methacryloyloxy)-*N*-(2-(methacryloyloxy)ethyl)-*N*,*N*-dimethylethan-1-aminium bromide
IDMA2	*N*,*N*′-([1,1′-biphenyl]-2,2′-diylbis(methylene))bis(2-(methacryloyloxy)-*N*,*N*-dimethylethan-1-aminium) bromide
MS	mass spectroscopy
n	number of specimens (or repetitive experiments)
NIR	near infra-red
NMR	nuclear magnetic resonance
P	phosphorus
PEG-U	poly(ethylene glycol) extended urethane dimethacrylate
pH	-log (molar concentration of hydrogen ions)
PO_4_	phosphate
PSD	particle size distribution
QADM	quaternary ammonium dimethacrylates
SD	standard deviation
SEM	standard error of the mean
TEGDMA	triethylene glycol dimethacrylate
UDMA	urethane dimethacrylate
UPE	UDMA/PEG-U/EHMA resin
XRD	X-ray diffraction
